# Isolation, characterization and *in vivo* efficacy of *Escherichia* phage myPSH1131

**DOI:** 10.1371/journal.pone.0206278

**Published:** 2018-10-24

**Authors:** Prasanth Manohar, Ashok J. Tamhankar, Cecilia Stalsby Lundborg, Nachimuthu Ramesh

**Affiliations:** 1 Antibiotic Resistance and Phage Therapy Laboratory, School of Bioscience and Technology, Vellore Institute of Technology (VIT), Vellore, Tamil Nadu, India; 2 Global Health-Health Systems and Policy (HSP): Medicines, focusing antibiotics, Department of Public Health Sciences, Karolinska Institutet, Stockholm, Sweden; 3 Indian Initiative for Management of Antibiotic Resistance, Deonar, Mumbai, India; Instituto Butantan, BRAZIL

## Abstract

Phage therapy is the use of lytic bacteriophages to cure infections caused by bacteria. The aim of this study is to isolate and to characterize the bacteriophages against *Escherichia coli* isolated from clinical samples. For isolation of bacteriophages, water samples were collected from the Ganges River, and phage enrichment method was followed for phage isolation. Microbiological, genomic and lyophilization experiments were carried out to characterize the bacteriophage. *Galleria mellonella* was used to study the potential of phages against *E*. *coli* infection. *Escherichia* phage myPSH1131 belonging to *Podoviridae* family and found to have broad host range infectivity (n = 31) to infect Enterohemorrhagic *E*. *coli* (n = 9), Enteropathogenic *E*. *coli* (n = 6), Enterotoxigenic *E*. *coli* (n = 3), Enteroaggregative *E*. *coli* (n = 3), Uropathogenic *E*. *coli* (n = 9) and one unknown *E*. *coli*. The genome size is 76,163 base pairs (97 coding regions) and their genes show high similarity to SU10 phage. Lyophilization studies showed that the use of 1M sucrose, 2% gelatin and the combination of both 0.5M sucrose plus 1% gelatin could restore phage viability up to 20 months at 4°C. For *in vivo* studies, it was observed that a single phage dose can reduce the *E*. *coli* infection but to achieve 100% survival rate the infected larvae should be treated with three phage doses (20 μL, 10^3^ PFU/mL) at 6 hours interval. The characterized *Escherichia* phage myPSH1131 was found to have broad host range activity against *E*. *coli* pathogens and *in vivo* studies showed that multiple doses are required for effective treatment.

## Introduction

Since the discovery of bacteriophages (phages), about a century ago, the use of phages for biological applications is increasing lately [1]. Bacteriophages are considered as a potent antibacterial agent because they are advantageous i.e. easy availability, naturally existing, specific in their activity and they can multiply rapidly in the presence of their host [1,2]. Bacteriophages are found to have a wider range of applications; in food processing industry, against plant pathogens, in water treatment plants, as a disinfectant for diagnostic tools and in therapy to treat bacterial infections [3,4]. Phage therapy is the therapeutic use of lytic bacteriophages for curing bacterial infections but the use of phages for the therapeutic purpose is minimal. The use of phages in therapy is limited to countries like Russia, Georgia and Poland [1,3]. Phage therapy is now getting renewed interest in the western medicine because of the developing antibiotic resistance infections [5]. With the use of modern technology, there are large numbers of research articles and clinical studies being performed to understand the role of phages as therapeutic agents. One of the biggest complications for phage therapy is the storage of phages for long-term or simply the half-life of phages is less than a year at 4°C. Though there are different approaches being developed such as encapsulation and aerosols, lyophilization (freeze-drying) of phages is considered the most efficient method [5,6]. The earlier studies that described the lyophilization of phages mainly focused on the type of stabilizers and the regimes used in freeze-drying. Further, the long-term storage (more than 20 months) of phages at lyophilized state will increase the use and application of phages in future.

*Escherichia coli* are also part of the normal microbiota but they are opportunistic pathogens causing urinary tract infections, diarrhoea, etc [7]. Because of the developing antibiotic resistance in bacteria, the use of antibiotics to cure *E*. *coli* infections are not effective nowadays. Therefore, effective alternative therapy is in demand to cure the infections caused by antibiotic-resistant *E*. *coli*. Phage therapy is considered one such therapeutic alternative to cure bacterial infections [8].

*Galleria mellonella* as an infection model is getting increasing popularity because of the advantages they provide over the other traditional mammalian models. *G*. *mellonella* larvae are easily available, cheaper than other model animals; easy maintenance at room conditions, short lifespan, and studies using them do not need ethical approval [9]. The earlier studies that used *G*. *mellonella* as an infection model for phage therapy studies showed promising outcomes and phages were shown to cure bacterial infections more effectively [10]. This study mainly focuses on the therapeutic characterization of isolated bacteriophage against *E*. *coli*, and to evaluate the stability of freeze-dried phages for long-term storage, and to test efficacy of bacteriophage using *G*. *mellonella* model.

## Materials and methods

### Ethical statement

This study was approved by Institutional Ethical Committee for Studies on Human Subjects—Vellore Institute of Technology Ref. no.: VIT/IECH/004/Jan28.2017.

### Collection of *E*. *coli* strains

For this study, the *E*. *coli* isolates were collected from diagnostic centers in Tamil Nadu (India) between December 2014 and March 2016. The clinical samples used for isolation included urine, pus, blood, sputum and bronchial waste. All the samples were processed at diagnostic center and the *E*. *coli* isolates were received at Antibiotic Resistance and Phage Therapy Laboratory, VIT, Vellore. The isolates were identified using VITEK identification system (bioMèrieux Inc., USA) and by molecular analysis using 16S rRNA analysis using the universal primers; 27F (5'-AGAGTTTGATCCTGGCTCAG-3') and 1492R (5'-GGTTACCTTGTTACGACTT-3'). All the clinical isolates used for this study were tested for antibiotic resistance pattern (carbapenem-resistant) using micro-broth dilution method (minimal inhibitory concentration) and for the identification of pathotypes (EPEC, EHEC, ETEC, EIEC, EAEC and UPEC), the primers and PCR conditions used were as explained elsewhere [11].

### Isolation and purification of bacteriophages

The bacteriophages were isolated from water samples collected from Ganges River near Varanasi region (as it is a natural source no permission is required to collect water samples) and transported under cold conditions (4°C) to Antibiotic Resistance and Phage Therapy Laboratory, VIT, Vellore. Phage enrichment method was used to isolate bacteriophages against the host bacterial strain (*E*. *coli* PSH131). Briefly, to the 3 mL of overnight grown bacterial cultures (host bacteria), 10 mL of water sample was added and the mixture was incubated at 37°C for 24 hours in a shaking incubator (150 rpm). The mixture was centrifuged at 6000 x g for 15 min and the supernatant was filtered through a 0.22-micron syringe filter. The filtrate was tested for phage activity using spot test and double agar overlay method. For the spot test method, to the bacterial lawn culture (host bacterium) prepared in the LB agar plate, 10 μL of phage filtrate was spotted. The plates were incubated at 37°C for 16–20 hours and the appearance of clear spots indicated the presence of bacteriophages. Briefly, in the double agar overlay method, 200 μL of host bacteria was mixed with 100 μL of phage filtrate and incubated for 15 min. To the mixture, 3 mL of molten soft agar was added, mixed and overlaid on to a hard agar plate. The plates were incubated for 16–20 hours at 37°C and the appearance of plaques showed the presence of a bacteriophage, and clear plaques indicated the lytic activity of phages. For phage precipitation, 10% polyethylene glycol MW6000 (PEG 6000) and 1M sodium chloride (NaCl) was added. The solution was mixed well (without vortex) and incubated at 4°C for 24 hours. The precipitated phage solution was centrifuged at 15,000 x g for 45 min and the pellet was diluted in SM buffer (For 1 L: 5.8g, NaCl; 50 mL, 1M Tris-HCl [pH 7.5]; 2g, MgSO_4_.7H_2_O; 5 mL, 2% gelatin). Further purification of phage particles for genomic analysis, CsCl gradient centrifugation and dialysis was performed as explained elsewhere [12].

### Host range determination

To determine the host range activity of phages; spot test, and double agar overlay methods were used [13]. Initially, the phages were isolated against the host bacterium (*E*. *coli* PSH131) and the broad host range activity of phage was assayed against the test bacteria (carbapenem-resistant *E*. *coli* strains (n = 53)). In spot test, to the bacterial lawn (test bacterium) the phage lysate (10^3^ PFU/mL) was spotted and the lytic activity was determined by the bacterial clearance on the phage spotted region. To further test the lytic activity of phages, double agar overlay method (quantitative assay) was performed for the test isolates that were having a zone of clearance in the spot test (as explained earlier). The number of plaques was counted against each test bacteria and compared with the host bacterium to determine the efficiency of plating (EOP). EOP was considered as ‘high’ when the difference in ratio between the host vs. test bacterium was <50 and others were considered as ‘low’. All the experiments were repeated in triplicates.

### Morphological characterization

To identify the morphological characteristics of phages, the phage particles were negatively stained using phosphotungstic acid (2% PTA) and visualized under transmission electron microscopy (TEM). Briefly, 10 μL of purified phages (10^5^ PFU/mL) were loaded on to a copper grid and allowed to dry for 10 min (excess liquid was removed). To the dried phage particles, 10 μL of 2% PTA was added, allowed for 5 min and the excess stain was removed. Finally, the stained copper grid was washed thrice with distilled water to remove the excess stain and viewed under TEM (FEI-TECNAI G2-20 TWIN, VIT, Vellore).

### One-step growth experiment

To determine the adsorption rate, the number of non-adsorbed phages was determined at a particular time interval [12]. Briefly, the bacterial culture (10^3^ CFU/mL, *E*. *coli* PSH131) was mixed with phages (MOI = 0.001) and incubated at 37°C. At every 4 min interval, an aliquot of 100 μL was removed from the mixture, diluted using LB broth (4.4 mL) and chloroform (0.5 mL). The aliquots were incubated for 30 min and the number of non-adsorbed phages was determined using the agar overlay method. To calculate the latent period and burst size, the phage particles (MOI = 0.001) were allowed to adsorb to the bacterial cells (10^8^ CFU/mL) and centrifuged at 10,000 x g for 5 min. To the pellet, 10 mL of LB broth was added and incubated at 37°C. The aliquots were removed at 5 min interval for 40 min and titrated against the host bacterium. The latent period is defined as the time taken for the phage particles to multiply/mature inside the bacterial cells i.e. from adsorption to first cell burst. Burst size is defined as the number of phage particles released from a single infected bacterial cell. All the experiments were repeated in triplicates.

### Stability studies

The thermal stability of phages was tested using 10^8^ PFU/mL of phage lysate that was subjected to different temperatures such as 4, 20, 35, 45, 50, 55, 60, 70, and 80°C for 60 min. After incubation in a temperature-controlled water bath, the phage activity was determined using the agar overlay method. In the case of pH, the ranges were from pH-1 to pH-14, and the phage lysate was incubated at the pH controlled environment for 60 min to determine the stability. The stability results were represented as percentage viability at different pH ranges. All the experiments were repeated in triplicates.

### DNA isolation, genome sequencing and analysis

The phage DNA was extracted from purified phage particles using phenol-chloroform (24:1) method and precipitated using ethanol (100%). Briefly, the purified phage particles were centrifuged at 10,000 x g for 10 min and the extracted supernatant was incubated with DNase (20 mg/mL) and RNase (0.5 mg/mL) for 1 h at 37°C. To the mixture, 20% PEG and 1.6M NaCl were added and stored for 1 h at 4°C. The content was centrifuged at 12,000 x g for 10 min and to the pellet, phage lysis buffer (50 μL of 10% SDA, 50 μL of 0.5M EDTA, and 5 μL of 10 mg/mL proteinase K) was added, mixed well and incubated overnight at 50°C. To the solution 600 μL of phenol: chloroform: isoamyl alcohol (25:24:1) was added and centrifuged at 12,000 x g for 15 min (repeated twice). The aqueous phase was extracted into a new tube and an equal volume of 100% isopropanol was added and allowed to precipitate at -20°C for 6 h. The precipitated DNA was centrifuged at 12,000 x g for 10 min at 4°C and the pelleted DNA was washed with 100% ethanol. The purified phage DNA was visualized on 0.8% agarose gels and the whole genome sequencing was performed using the Illumina Nextseq 500 system at Eurofins Genomics India Pvt Ltd. The sequenced raw data was processed to obtain high-quality clean reads using Trimmomatic v0.35 to remove adapter sequences, ambiguous reads (reads with unknown nucleotides “N” larger than 5%), and low-quality sequences (reads with more than 10% quality threshold (QV) < 20 phred score). The sequenced high-quality reads were *de novo* assembled using CLC Genomics Workbench version 9.5.2. Protein-coding and tRNA genes were identified using the final assembly. The transfer-RNA (tRNA) genes were predicted using tRNAscan-SE 2.0 web server while the protein-coding genes (CDS) were predicted using FGENESV web server. Functional annotation of the predicted proteins was performed using the amino acid sequences via BLASTp program online against a custom database of viral proteins in NCBI. Gene ontology (GO) annotations of the genes were determined by the Blast2GO platform. Distribution of GO terms across the categories–Biological Process, Molecular Function and Cellular Component was obtained through WEGO portal (http://wego.genomics.org.cn/cgi-bin/wego/index.pl). The NCBI sequence was downloaded from NCBI (https://www.ncbi.nlm.nih.gov/nuccore/KJ101592) for sequence comparison and the scaffolds were then subjected to the *de novo* assembly via CONTIGuator2. The final assembly generated by CONTIGuator was validated based on sequence homology to known bacteriophage sequences in NCBI via BlastN. The complete genome was submitted to NCBI (BankIt) and accession number was generated.

### Lyophilization

For phage freeze-drying, the lyophilization conditions used were; initial cooling of the sample holding shelves to 5°C, whereas samples were precooled to -20°C. Once the samples were loaded, the shelves were cooled to -30°C at 1°C/min for 90 min. During primary drying the samples were maintained at -30°C for 12 h (100 millitorr) and in secondary drying the temperature was raised from -30°C to 25°C at 0.1°C/min for 10 h (100 millitorr) [14]. The phage samples were prepared using six stabilizers and their combinations for freeze-drying. The stabilizers used were; glucose (0.5M and 1.0M), sucrose (0.5M and 1.0M), mannitol (0.5M and 1.0M), gelatin (1% and 2%), PEG 6000 (0.1M and 0.5M), sorbitol (0.5M and 1.0M), 1.0 M glucose plus 1% gelatin and 0.5M sucrose plus 1% gelatin. One milliliter of phage solution was prepared using 300 μL of phage lysate (5x10^8^ PFU/mL) and 700 μL of the stabilizer. The phage solution was prepared in 10 mL lyophilizing vials and loaded on to a Benchtop K freeze dryer (VirTis, US). The freeze-dried phage products were sealed and stored at -20°C for further analysis. After lyophilization, the phage particles were resuspended using SM buffer [5.8g NaCl; 50 mL 1M Tris-HCl [pH 7.5]; 2g MgSO_4_.7H_2_O; 5 mL; 0.5M sucrose; 1% gelatin for 1000 mL]. To calculate the phage viability after lyophilization, the initial phage titer (before lyophilization) and final phage titer (after lyophilization) was evaluated. The viability of lyophilized phages through long-term storage was determined by storing the freeze-dried phages at 4°C up to 20 months. The half-life of the lyophilized phages was determined using the double agar overlay method for 20 months upon testing at every five months interval. The storage stability of lyophilized phages was compared with phages stored as suspensions under the same conditions (at 4°C for 20 months). The data were interpreted based on the results obtained from three independent experiments.

### Bacterial infection and treatment of *G*. *mellonella* using single and multiple phage doses

For this study, *G*. *mellonella* larvae were collected from Department of Entomology, University of Agricultural Sciences, Gandhi Krishi Vignana Kendra, Bengaluru, India. The worms chosen for the study were app. 2–2.5 cm and creamy white in color. For larval infection, the bacterial inoculum of 10^6^ CFU/mL and the phage titer at 10^3^ PFU/mL was injected into the last pair of pro-left legs [15]. For each group, 15 worms were used for the study and the worms were starved for 24 hours before starting the experiment. The study was performed as follows; in Group-1, 20 μL of saline was injected and larval movement was evaluated to reduce the injury during injection; in Group-2, 20 μL of bacteria (alone) was injected and the virulence was evaluated at 12, 24, 48, 72 and 96 hours; in Group-3, 20 μL of phage lysate (alone) was injected and larval survival was evaluated for toxicity; in Group-4, initially larvae were infected with 20 μL of bacterium and within 1 hour they were treated with 20 μL of phage lysate (single dose) and the larval survival was evaluated at 12, 24, 36, 48, 60, 72 and 96 hours. In order to evaluate the effect of multiple phage doses on larval recovery, four groups with four doses were maintained; Set-1: the larvae were infected with bacteria and the first dose was given at 0^th^ hour, Set-2: the infected larvae were treated with two doses of phages at 0^th^ and 6^th^ hour, Set-3: the infected larvae were treated with three doses of phages at 0^th^, 6^th^ and 12^th^ hour, Set-4: the larvae received four phage doses at 0^th^, 6^th^, 12^th^ and 24^th^ hour. The worms were considered as alive only when the worms were moving freely without any stimulation or no melanization and others were considered as dead. All the results were expressed as percentage (%) survival of larvae at different time intervals and all the experiments were performed in triplicates. To determine the reduction in the bacterial count during phage therapy, the larval faecal samples were collected and enumerated for the bacterial load. The bacterial enumeration from dead and live larvae was performed on LB agar plates (0.1 mL) and bacterial load of CFU per larva was evaluated. Five larvae from each group were examined and all the experiments were performed in triplicates and analysed using GraphPad Prism 7.0.

### Statistical analysis

GraphPad Prism 7.0 software was used to express the study results. In the case of survival curves, Kaplan-Meier method and a *log-rank test* were used to calculate the difference in survival rates using GraphPad Prism 7.0 software (GraphPad Software, Inc., La Jolla, USA). P < 0.05 was considered statistically significant.

## Results and discussion

### Characterization of *Escherichia* phage myPSH1131

**Fig 1:**
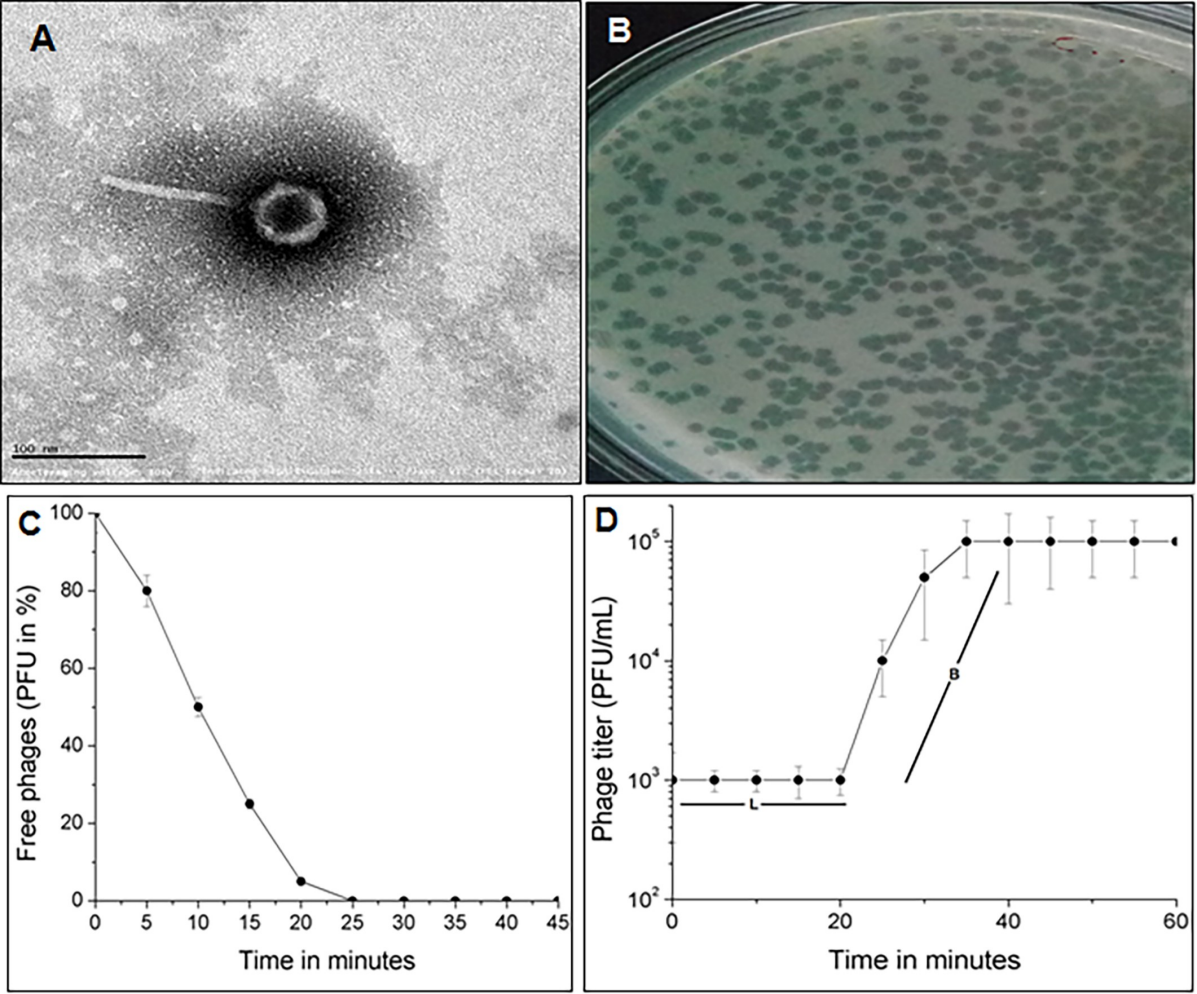
Morphological characterization and life cycle of *Escherichia* phage myPSH1131. A) Transmission Electron Microscopic (TEM) image of *Escherichia* phage myPSH1131 shows that the phage belongs to *Podoviridae* family, B) Observed plaques of *Escherichia* phage myPSH1131 infecting *E*. *coli* PSH131 using double agar overlay method, C) Adsorption velocity of *Escherichia* phage myPSH1131 showing the adsorption velocity of 1.6x10^-9^ mL/min, D) One-step growth curve of *Escherichia* phage myPSH1131 showing the latency period (L) of 20 min and burst size (B) of 130 phage particles/infected cell.

For this study, a total of 53 *E*. *coli* isolates were collected from the Hi-Tech diagnostic center, Tamil Nadu, India. The isolates were identified to be *E*. *coli* by 16S rRNA analysis and pathotyping results showed that the *E*. *coli* isolates (n = 53) belonged to EPEC (n = 13), EHEC (n = 10), ETEC (n = 5), EIEC (n = 3), EAEC (n = 6), UPEC (n = 12) and 4 of unknown pathotype. All the 53 isolates were found to be resistant to meropenem (carbapenem-resistant) and included to study the host-range activity of bacteriophages. *Escherichia coli* are a well-known pathogen causing opportunistic infections such as diarrhoea, fever, urinary tract infections in both children and adults [16]. A total of two bacteriophages were isolated in this study, of which one phage that showed broad host range activity was further characterized. The isolated phage was named as *Escherichia* phage myPSH1131 according to bacteriophage naming guidelines provided by Adriaenssens and Brister, 2017. The phage morphology was determined by TEM analysis and it showed that the *Escherichia* phage myPSH1131 belonged to *Podoviridae* family having the icosahedral head of approximately 45±5.0 nm and long non-contractile tail of approximately 95±5.0 nm in length (Fig 1A). The earlier studied *Escherichia* phages of *Podoviridae* family are SU10 [17], podovirus [18], IME-EC2 [19]. Microscopic images showed that the *Escherichia* phage myPSH1131 has some structural similarities to *Escherichia* phage SU10, phiEco32 and NJ01, and later it was also proven by genomic similarities within these phages. *Escherichia* phage is studied extensively in different parts of the world because of the severity of the *E*. *coli* infections and there is a large scale use of *Escherichia* phage cocktails at phage therapy centers in Russia and Georgia [20].

### Host-range activity

For determining the host-range activity, the initial spot test results showed that, of the 53 *E*. *coli* isolates (carbapenem-resistant) tested, the *Escherichia* phage myPSH1131 was active (lytic activity) against 31 isolates that belonged to EPEC (n = 9), EHEC (n = 6), ETEC (n = 3), EAEC (n = 3), UPEC (n = 9) and one of unknown pathotype (Table 1). Earlier it was reported that the activity of phages (host specificity) may vary depending upon the type of method used for host range determination and also upon the efficiency of phages [13]. To further evaluate the host range infectivity, double agar overlay method (Fig 1B) was performed that showed that the *Escherichia* phage myPSH1131 infected (formed plaques) 29/53 *E*. *coli* isolates tested. Though spot test results showed *Escherichia* phage myPSH1131 had clear spots against 31 *E*. *coli* isolates, the agar overlay method showed plaques against 29 *E*. *coli* isolates which clearly exhibited the specificity of each method. The EOP analysis data showed high/ good productive infection (differences in plaques formed between the host vs. test bacterium was <50) against 14/29 *E*. *coli* isolates, low/ poor infectivity (at least one plaque) was found against 15/29 *E*. *coli* isolates and no infection against 2 *E*. *coli* isolates (Table 1). The important aspect of *E*. *coli* as a pathogen is their various serotypes and pathotypes, so the choice of phages for therapy should have a broad spectrum of activity against different serotypes/ pathotypes of *E*. *coli*. Earlier, a study by Bourdin *et al*., reported the use of phage cocktails (prepared using 89 T4-like phages) to achieve the broad-host-range infection in *E*. *coli* and the study stated that there was no broad-host-range in any of the studied phage [16]. There are several studies that reported the use of phage cocktails for the treatment of *E*. *coli* infections [21–26] and >75 *Escherichia* phage genome sequences are available in NCBI (https://www.ncbi.nlm.nih.gov/genome). In this study, the isolated *Escherichia* phage myPSH1131 showed lytic activity against 5 different pathotypes that clearly showed the broad host range activity of this phage. The *Escherichia* phage myPSH1131 was found to share similarity with SU10 and phiEco32 phages in their morphological and genomic characteristics that may prove that *Escherichia* phage myPSH1131 has no impact on commensal microbiota (17). Our study is one of the rarest studies of this kind that demonstrated the broad-host-range activity of a single *Escherichia* phage (*Escherichia* phage myPSH1131) infecting 5 different pathotypes of *E*. *coli* isolated from Tamil Nadu, India.

**Table 1:** Host-range analysis results of *Escherichia* phage myPSH1131 against carbapenem-resistant *E*. *coli* strains. Results of spot test assays and EOP (efficiency of plating) analysis on *E*. *coli* strains belonging to different pathotypes.

Bacterial isolate	Isolation source	Pathotype	Spot test	EOP
*E. *coli* PSH131	Blood	EPEC	+	High
*E*. *coli* PSH100	Blood	EHEC	-	-
*E*. *coli* PSH103	Sputum	EIEC	-	-
*E*. *coli* PSH110	Pus	EPEC	-	-
*E*. *coli* PSH123	Sputum	unknown	-	-
*E*. *coli* PSH150	Blood	EHEC	-	-
*E*. *coli* PSH151	Bronchial waste	ETEC	-	-
*E*. *coli* PSH167	Sputum	EIEC	-	-
*E*. *coli* PSH200	Pus	EPEC	+	Low
*E*. *coli* PSH201	Blood	EPEC	+	Low
*E*. *coli* PSH206	Sputum	EPEC	+	High
*E*. *coli* PSH219	Sputum	EIEC	-	-
*E*. *coli* PSH233	Urine	UPEC	-	-
*E*. *coli* PSH241	Pus	EPEC	+	Low
*E*. *coli* PSH249	Blood	ETEC	-	-
*E*. *coli* PSH256	Sputum	EPEC	+	Low
*E*. *coli* PSH260	Blood	EPEC	+	High
*E*. *coli* PSH261	Sputum	EPEC	+	High
*E*. *coli* PSH263	Blood	EHEC	-	-
*E*. *coli* PSH264	Sputum	EAEC	-	-
*E*. *coli* PSH268	Pus	EPEC	+	Low
*E*. *coli* PSH276	Sputum	unknown	-	-
*E*. *coli* PSH280	Urine	UPEC	-	-
*E*. *coli* PSH290	Blood	EHEC	+	Low
*E*. *coli* PSH297	Sputum	EAEC	-	-
*E*. *coli* PSH299	Blood	EHEC	+	Low
*E*. *coli* PSH301	Blood	EHEC	+	High
*E*. *coli* PSH303	Blood	EHEC	+	High
*E*. *coli* PSH206	Sputum	EAEC	-	-
*E*. *coli* PSH311	Blood	EHEC	+	High
*E*. *coli* PSH315	Blood	EHEC	+	High
*E*. *coli* PSH320	Urine	UPEC	-	-
*E*. *coli* PSH324	Bronchial waste	ETEC	+	High
*E*. *coli* PSH333	Sputum	ETEC	+	High
*E*. *coli* PSH335	Blood	ETEC	+	Low
*E*. *coli* PSH250	Sputum	EPEC	-	-
*E*. *coli* PSH356	Blood	EAEC	+	Low
*E*. *coli* PSH360	Sputum	EAEC	+	High
*E*. *coli* PSH365	Sputum	EAEC	+	Low
*E*. *coli* PSH366	Urine	UPEC	+	High
*E*. *coli* PSH367	Urine	UPEC	+	High
*E*. *coli* PSH271	Bronchial waste	unknown	-	-
*E*. *coli* PSH389	Urine	UPEC	+	Low
*E*. *coli* PSH390	Urine	UPEC	+	Low
*E*. *coli* PSH391	Sputum	EPEC	-	-
*E*. *coli* PSH395	Blood	EHEC	-	-
*E*. *coli* PSH399	Urine	UPEC	+	NA
*E*. *coli* PSH401	Bronchial waste	EPEC	-	-
*E*. *coli* PSH411	Urine	UPEC	+	High
*E*. *coli* PSH413	Urine	UPEC	+	Low
*E*. *coli* PSH414	Urine	UPEC	+	Low
*E*. *coli* PSH415	Urine	UPEC	+	Low
*E*. *coli* PSH416	Urine	unknown	+	NA

EOP = Efficiency of plating and determined using double agar overlay method, phages at 10^3^ PFU/mL was used for the study

* represents host bacterium; NA = no activity; + = phage active; EOP was considered as ‘high’ when the difference in ratio between the host vs. test bacterium was ≥50 and <50 was considered as ‘low’.

### One-step growth experiment

The life cycle of bacteriophages is one of the important criteria to evaluate the lytic activity and to determine the infectivity of therapeutic bacteriophages. Accordingly, adsorption rate, latency time and burst size were determined by one-step growth experiment in the presence of host *E*. *coli* PSH131. The *Escherichia* phage myPSH1131 was found to have an adsorption velocity of 1.6x10^-9^ phage/cell/mL/min, a latency time of 20 min and the calculated burst size was 130 ± 10 phage particles/ infected cell (Fig 1C and 1D). The short latent period (time for multiplication of phages within the host) and the huge amount of phage release from the infected host (burst size) clearly showed that the *Escherichia* phage myPSH1131 was a lytic phage having a very adapted therapeutic potential. Similarly, latent period and burst size were observed in other *E*. *coli* phages SU10 and phiEco32 belonging to *Podoviridae* family (17). An earlier study by Dalmasso *et al*. reported the population kinetics of three *E*. *coli* bacteriophages, ϕAPCEc01, ϕAPCEc02 and ϕAPCEc03 belonging to *Myoviridae* and *Siphoviridae* having the latent period of 60 min, 40 min and 40 min with a burst size of 90, 30 and 47 phage particles respectively [27]. Bacteriophages are able to lyse their hosts at temperatures between 20–37°C and are inactivated only at temperatures >60°C and at pH <2 or >13 [28]. The stability studies showed that the *Escherichia* phage myPSH1131 was stable between the pH range of 3 to 13 and a significant reduction in phage activity was observed at pH <3 and >13. The selection of phages that are stable at lower pH is important for *E*. *coli* infections because of the acidic nature of the intestine where most of the *E*. *coli* infections occur. The results suggested that the *Escherichia* phage myPSH1131 was tolerant to both acidic and basic conditions. No loss in phage activity (titer) was observed from 4 to 45°C but a titer loss was observed at 50°C and above.

### General features of phage genome

The genome of *Escherichia* phage myPSH1131 was 76,163 bp in size and the GC content of this phage was 42.3% (A-28.5%, T-29.1%, G-20.2% and C-21.9%). It was found to have 97 coding regions (89.3% ORFs), includes 31 proteins of known putative function and 66 hypothetical proteins. Assembly report showed that many genomic features of *Escherichia* phage myPSH1131 having a high resemblance to the genome of SU10 and phiEco32. For instance, the G+C content 42.3% (*Escherichia* phage myPSH1131), which was very close to the 42.1% and 42.3% G+C content of SU10 and phiEco32 respectively. The sequencing and gene annotation report showed that the clustered arrangement of genes which was grouped as; 12 genes with biological processes, 4 genes with cellular component and 15 genes with molecular function. The phage genome was not found to have any toxins or toxin-related genes and none of the genomic markers representing a temperate or lysogenic lifestyle was found (Table A in S1 File). These characteristics make *Escherichia* phage myPSH1131 suitable for phage therapy.

**Fig 2:**
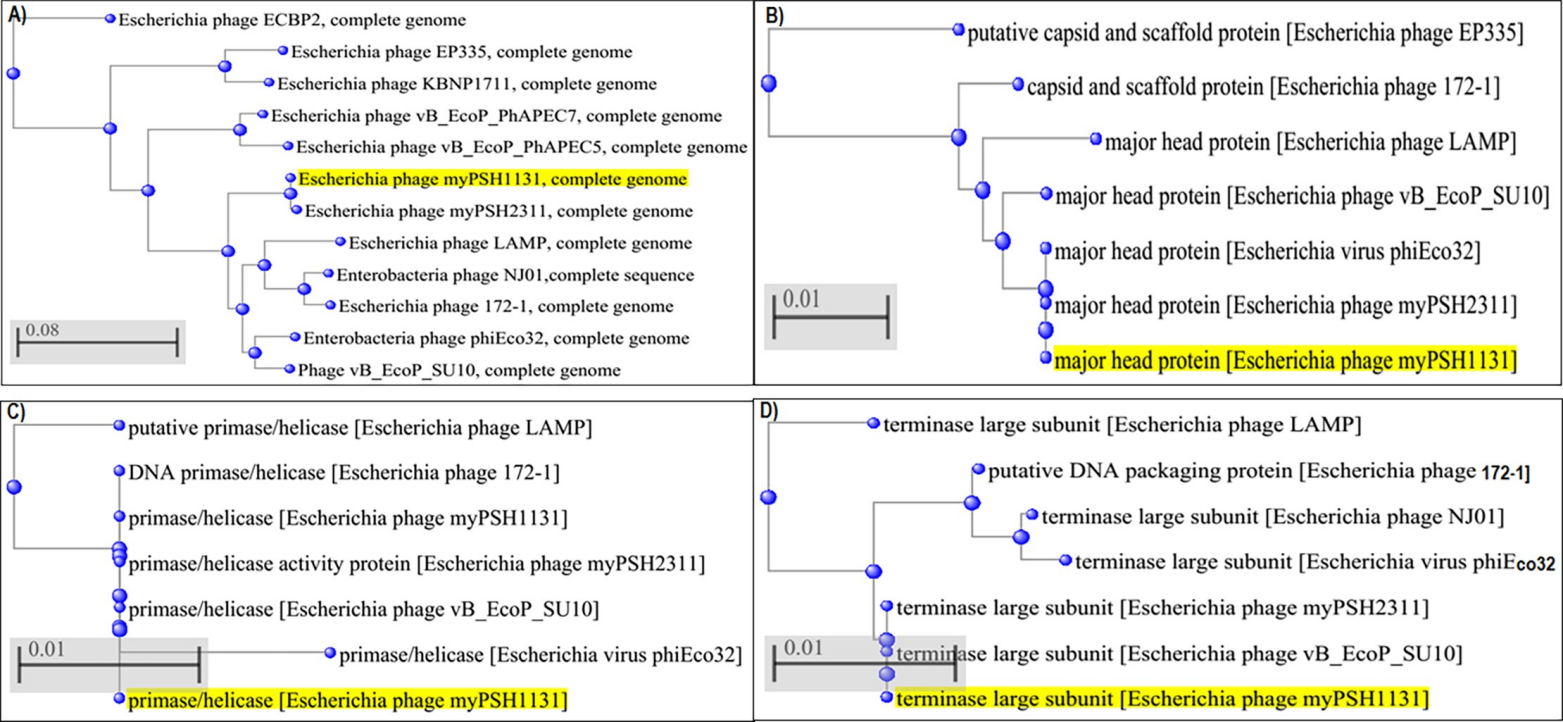
Phylogenetic tree analysis of *Escherichia* phage myPSH1131 and their proteins based on genome sequences of selected phages in NCBI-BLAST. A) Complete genome, B) Major head protein, C) Primase/helicase protein, D) Terminase protein. The tree was produced using BLAST pairwise alignment using the Neighbor-Joining method. The query sequence is highlighted in yellow.

**Fig 3:**
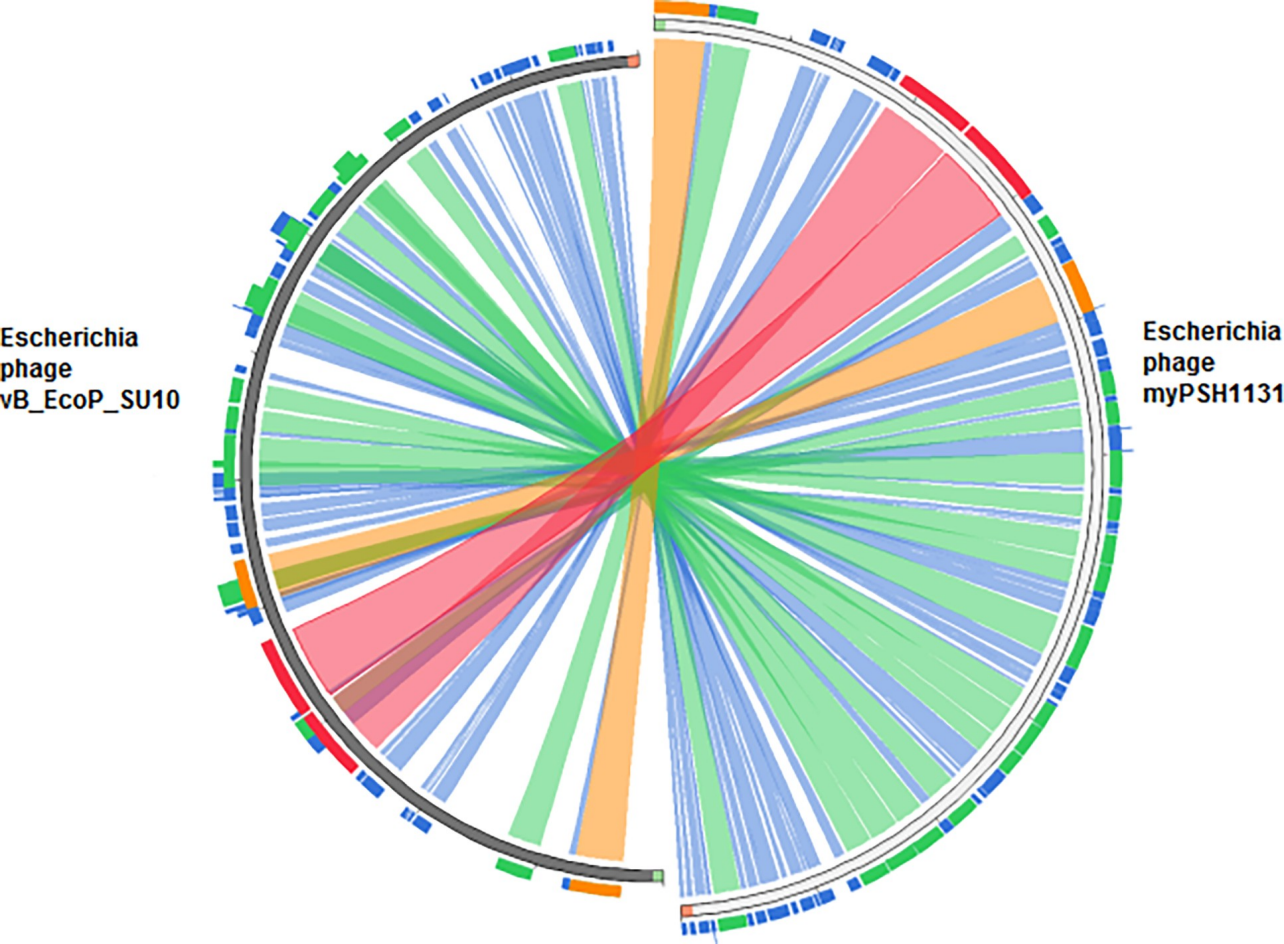
Genome sequence similarity of *Escherichia* phage myPSH1131 and *Escherichia* phage vB_EcoP_SU10. The nucleotide sequence of *Escherichia* phage myPSH1131 and *Escherichia* phage vB_EcoP_SU10 was compared and found to have 59% nucleotide sequence similarity. Similarity index: The red color signifies high sequence similarity followed by orange, green and blue.

The arranged complete genome of *Escherichia* phage myPSH1131 is closely related at the nucleotide level to the other *Escherichia* phages with C3 morphology (*Escherichia* phage vB_EcoP_SU10 and *Escherichia* virus phiEco32) (Fig 2). *Escherichia* phage myPSH1131 shares 59% identity with *Escherichia* phage vB_EcoP_SU10 (Fig 3). *Escherichia* phage vB_EcoP_SU10 was documented to have a genome of 77,327 bp, linear and with 125 coding sequences (CDS) (Figure A in S1 File). Other closely related phage genomes were *Escherichia* virus phiEco32 with 47% identity, *Escherichia* phage 172–1 with 29% identity and *Escherichia* phage NJ01 with 26% identity (Fig 2). The conserved ORF regions in *Escherichia* phage myPSH1131 such as head and tail proteins, polymerases and terminases show high identity (80–100%) with *Escherichia* phage vB_EcoP_SU10. However, there are differences observed in gene content of other similar phages (Table A in S1 File). The conserved lysin protein in *Escherichia* phage myPSH1131 may enhance the broad-host-range activity and also the efficacy of using this phage in therapeutic applications.

### Stability of lyophilized phages

**Fig 4:**
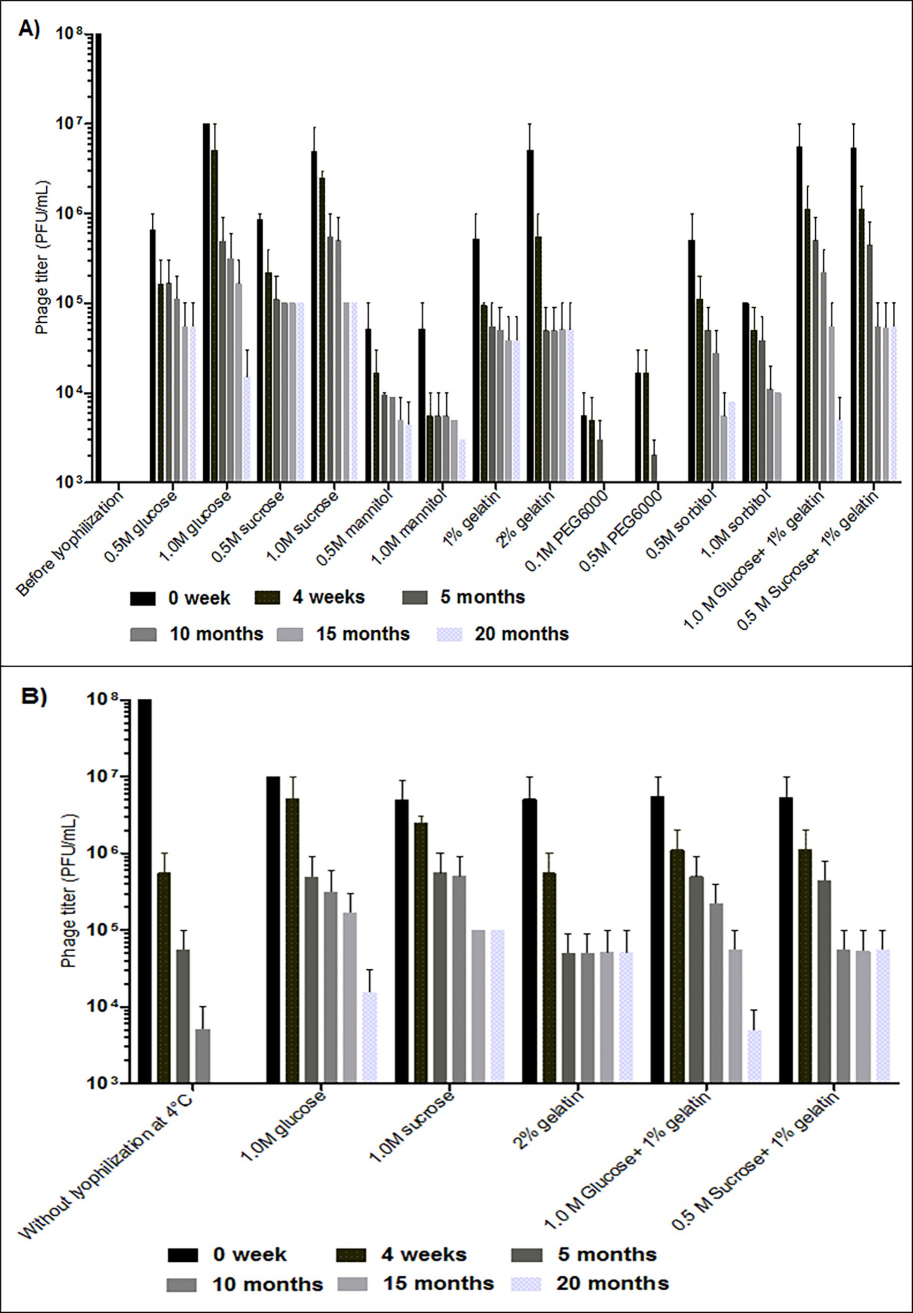
Estimate of long-term storage stability of *Escherichia* phage myPSH1131. A) Stability of lyophilized (freeze-dried) *Escherichia* phage myPSH1131 using six different stabilizers and their combinations, and B) Comparison of lyophilized *Escherichia* phage myPSH1131 with the phages stored as suspensions at 4°C. The initial titer of phage was 10^8^ PFU/mL (before lyophilization) and the represented results are mean values of three independent experiments.

The *Escherichia* phage myPSH1131 was found to be a good candidate phage for therapeutic purpose so lyophilization properties of this phage was further studied. The advantages of having lyophilized phages are that they can withstand for long-term storage (increased half-life) and can be used in different pharmaceutical formulations [5]. The preparation of lyophilized (freeze-dried) phages to increase the viability of phages typically involves the selection of stabilizers for lyophilization and rehydrating solutions used for their recovery. In this study, six stabilizers and their combinations were tested against the *Escherichia* phage myPSH1131 that showed the stabilizers 1M glucose, 1M sucrose, 2% gelatin and 0.5M sucrose plus 1% gelatin was able to retain the phage activity after lyophilization (Fig 4A). Nowadays, sugars are considered as good cryoprotectants because of their ability to form glass matrix during freezing that could protect the lyophilized particles from aggregation [5]. Gelatin was found to have a strong influence on phage lyophilization when mixed with SM buffer [14] but to the best of our knowledge, this is the first study to report the use of gelatin as a stabilizer for phage lyophilization. The important aspect of this study is the use of sugar plus gelatin combination as a stabilizer, whereas, only a titer of phage reduction was observed after lyophilization (Fig 4A). The other sugars such as mannitol and sorbitol showed poor phage stabilizing properties, and PEG6000 showed >4 log reduction in phage titer after lyophilization. An earlier study by Merabishvili *et al*., also showed that PEG6000 and mannitol as poor stabilizers [5]. After lyophilization, the lyophilized phages were stored at 4°C to evaluate the long-term storage (20 months) stability. The results showed that the phages that were lyophilized using 1M sucrose, 2% gelatin and 0.5M sucrose plus 1% gelatin showed stable activity up to 20 months with <2 log reduction in phage titer (Fig 4A). The results also showed SM buffer as a good rehydrating agent after long-term storage of lyophilized phages. When phages were stored as suspensions the viability of the phages was decreased drastically after 4 weeks at 4°C (Fig 4B), clearly showing the importance of phage lyophilization for long-term storage of bacteriophages. Our study did not compare the use of different rehydrating solutions in restoring phage titer for long-term storage and the studied lyophilized phages were stored only at 4°C and the stability was not tested at room temperature (37°C). But earlier studies proved the ability of SM buffer as a good cryoprotectant for phage storage [14]. There are various studies on phage lyophilization that used different stabilizers to store the phages for the longer period but for the therapeutic utility of phages the studies in future may have to consider the choice of stabilizers that can be used as pharmacological formulations. Therefore, in this study, the stabilizers/cryoprotectants chosen were pharmaceutically acceptable (only sugars) that can be used for future applications.

### *In vivo* efficiency of single and multiple phage doses in *G*. *mellonella* model

**Fig 5:**
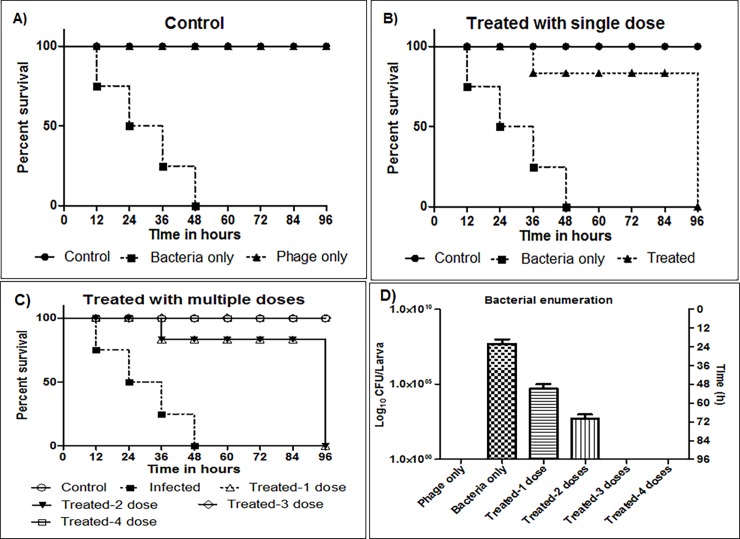
Impact of *Escherichia* phage myPSH1131 treatment on *G*. *mellonella* larval survival. **A**) Control group: The larvae was injected with saline and phage only (10^3^ PFU/mL), and infected with bacteria (10^6^ PFU/mL).**B**) Impact of single dose phage treatment on *E*. *coli* PSH131 and survival rates of *G*. *mellonella* larvae. A single phage dose (10^3^ PFU/mL) was injected at 0^th^ hour after the larvae was pre-infected with 10^6^ CFU/mL of bacteria. Survival rates of the larvae infected with *E*. *coli* PSH131 treated with single dose of *Escherichia* phage myPSH1131 (P = 0.0045) **C**) Impact of multiple phage doses: The multiple phage doses (10^3^ PFU/mL) were injected at 0^th^, 6^th^, 12^th^ and 24^th^ hour after the larvae were pre-infected with 10^6^ CFU/mL of bacteria. The survivability of larvae infected with *E*. *coli* PSH131 and treated with *Escherichia* phage myPSH1131 (<P = 0.0001) **D**) Bacterial enumeration from larvae, results are represented as CFU of bacteria/larva The survival rates were plotted using the Kaplan-Meier method and *log-rank test* was used to analyze the difference in survival rates. A statistically significant difference (P < 0.05) was observed using 15 worms per group on phage treatment. Error bars represent standard error of the mean (SEM) of three independent replicates and data were analyzed using GraphPad Prism 7.0.

The *in vivo* activity of the *Escherichia* phage myPSH1131 was studied using *G*. *mellonella* as a model organism. When the worms were injected with saline and the phage lysate (20 μL), 100% survivability was observed that clearly showed that there was no injury during injection as well as there were no toxic byproducts in the phage lysate that could kill the larvae. To study the bacterial pathogenesis, 10^6^ CFU/mL of *E*. *coli* PSH131 was injected and the worms were found to be dead within 48h (Fig 5A). Initially, when single dose phage treatment was used, there was 100% survival up to 24h and a gradual decrease in the larval movement was observed after 36h (Fig 5B). Because of the severity of bacterial infection, a single phage dose treatment was not sufficient to achieve 100% larval survival. In order to evaluate the efficiency of multiple phage doses, up to four doses of phages were used to treat the infected larvae. Accordingly, during the first two doses of phage treatment the recovery was very minimal with <75% survivability after 36h and bacterial enumeration using larval faecal matter also showed the viable bacterial count. But during the 3^rd^ and 4^th^ phage dose treatments, there was 100% larval survival (Fig 5C) and similar results were observed during the bacterial enumeration (Fig 5D). Though the *Escherichia* phage myPSH1131 was found to have a shorter life cycle and the phages are naturally self-replicating, our study showed that a single phage dose treatment was not sufficient to eradicate the bacterial load completely. Our results clearly showed that the choice of treatment and the dosage of phages should be under consideration during therapeutic application of phages. For the better therapeutic outcome, the route of administration of phages will have a profound effect. A single phage dose treatment was found to be less effective because of the rapid multiplication of bacteria that worsened the larval condition within 72h. These complications should be avoided during phage therapy by adopting multiple phage doses instead of single complex doses that could lead to rapid release of lipopolysaccharides (LPS) and other side effects. This study showed promising outcomes in *G*. *mellonella* infection model when multiple phage doses were used. An earlier study by Seed *et al*., showed the increased survival rates in *G*. *mellonella* that are infected with *Burkholderia cepacia* after treatment with bacteriophages [29], and other recent studies showed the therapeutic use of *E*. *coli* phages against mammalian models [30]. A simple animal model like wax worm can serve as a tool to determine the initial outcome of the phages in reducing bacterial infections. The number of studies that used *G*. *mellonella* as a model organism is rare [29,31] so to the best of our knowledge this is the first study to report the therapeutic potential of *Escherichia* phage against *E*. *coli* infections using *G*. *mellonella* as a model. Though our study showed promising outcomes for the use of *Escherichia* phage myPSH1131 against *E*. *coli* PSH131 infections using a simple animal model (wax moth larvae), in future more detailed studies are needed to understand the dynamic relationship between host bacterium and bacteriophages.

## Conclusion

The isolated *Escherichia* phage myPSH1131 against pathogenic *E*. *coli* was found to be effectively eliminating or reducing the bacteria in both *in vitro* and *in vivo*. The *Escherichia* phage myPSH1131 was found to have broad host range infectivity against five different pathotypes of *E*. *coli* which is one of the important aspects for the selection of phages during therapy. Lyophilization studies showed that the use of sucrose and the combination of sucrose plus gelatin can restore the viability of *Escherichia* phage myPSH1131 during long-term preservation at 4°C. *In vivo* studies using *G*. *mellonella* as a model organism showed promising results that can be considered in the future for testing in murine models and for clinical trials. Antibiotic resistance has attained a dangerous level to the humankind that needs to be addressed with an alternative option such as phage therapy. Relentless efforts are being taken to study the science behind phage therapy and our study is one such an effort to improve the understanding behind the use of bacteriophages as a therapeutic tool.

## Supporting information

S1 File **Figure A. Comparative analysis of whole genome sequences using wgVISTA database**. Depicts the sequence similarities of *Escherichia* phage myPSH1131 against *Escherichia* phage vB_EcoP_SU10. **Table A. *Escherichia* phage myPSH1131 genome annotation**.
(DOCX)
