# Supplementary material for: Isolation, characterization and in vivo efficacy of Escherichia phage myPSH1131
Source: PLoS One. 2018 Oct 24;13(10):e0206278. doi: 10.1371/journal.pone.0206278 (PMC6200275; doi:10.1371/journal.pone.0206278)
Supplement: S1 File — Figure A. Comparative analysis of whole genome sequences using wgVISTA database. Depicts the sequence similarities of Escherichia phage myPSH1131 against Escherichia phage vB_EcoP_SU10. Table A. Escherichia phage myPSH1131 genome annotation. (DOCX) [file pone.0206278.s001.docx]

**Figure A: Comparative analysis of whole genome sequences using wgVISTA database.** Depicts the sequence similarities of *Escherichia* phage myPSH1131 against *Escherichia* phage vB_EcoP_SU10.

**
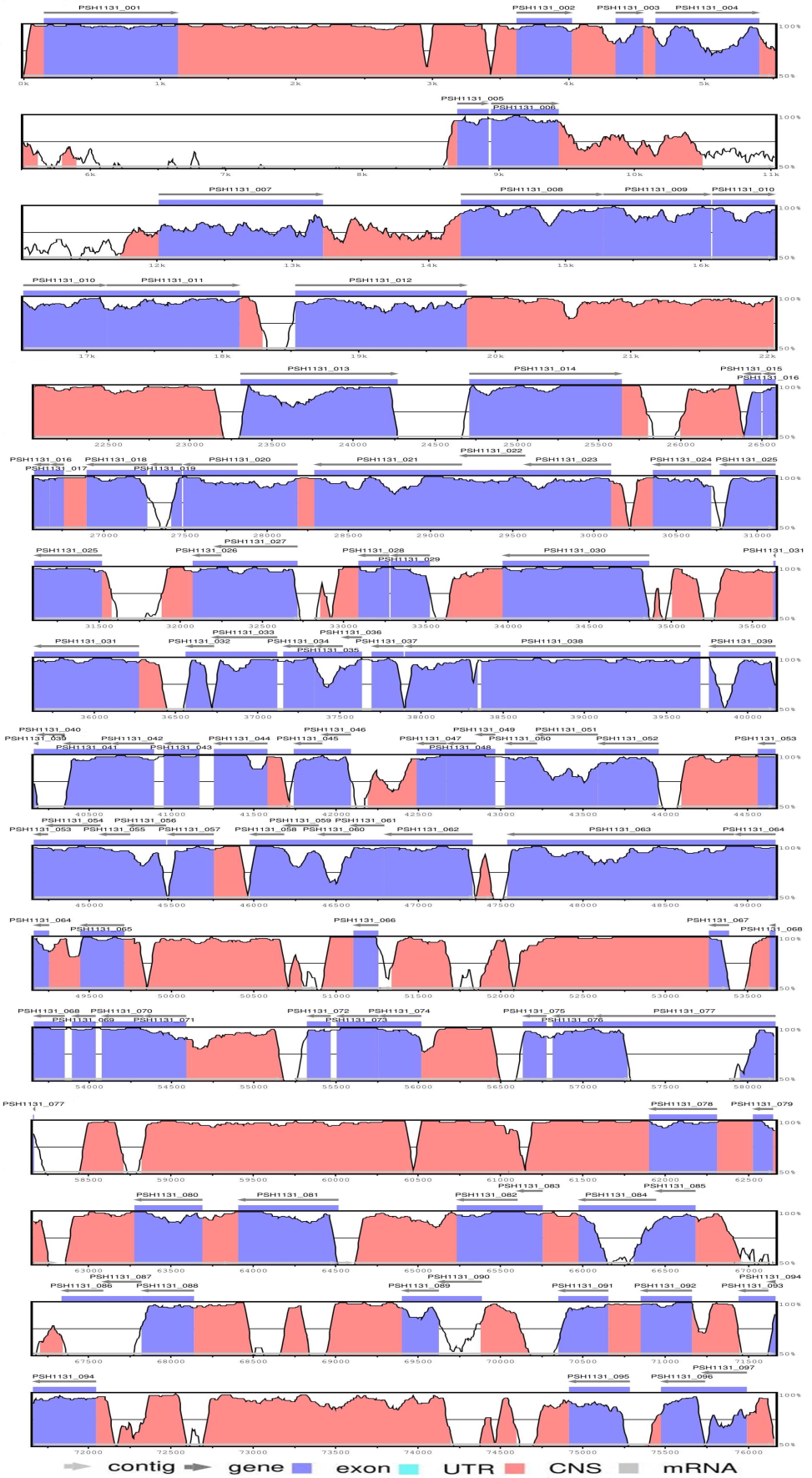
**

**Table A: *Escherichia* phage myPSH1131 genome annotation.**

| **ORFs** | **Nucleotide Position** | **Strand** | **Function** |
| --- | --- | --- | --- |
| ORF1 | 151-1131 | **+** | **Terminase large subunit** |
| ORF2 | 3622-4020 | **+** | **Major head protein** |
| ORF3 | 4350-4544 | **+** | **Bacterial Ig-like domain** |
| ORF4 | 4647-5399 | **+** | Hypothetical protein |
| ORF5 | 8701-8919 | **+** | **Holin** |
| ORF6 | 8949-9440 | **+** | **Lysin** |
| ORF7 | 12021-13223 | **+** | **Surface protein** |
| ORF8 | 14244-15275 | **+** | Hypothetical protein |
| ORF9 | 15288-16070 | **+** | **Internal virion D** |
| ORF10 | 16090-17142 | **+** | Hypothetical protein |
| ORF11 | 17155-18123 | **+** | **DNA injection protein** |
| ORF12 | 18536-19786 | **+** | Hypothetical protein |
| ORF13 | 23310-24263 | **+** | Hypothetical protein |
| ORF14 | 24709-25635 | **+** | **DNA injection** |
| ORF15 | 26387-26494 | **-** | Hypothetical protein |
| ORF16 | 26507-26668 | **-** | Hypothetical protein |
| ORF17 | 26677-26757 | **-** | Hypothetical protein |
| ORF18 | 26897-27265 | **-** | **Terminase small subunit** |
| ORF19 | 27385-27476 | **-** | Hypothetical protein |
| ORF20 | 27490-28182 | **-** | **5’-3’ exonuclease** |
| ORF21 | 28295-29188 | **-** | **ATP-binding protein** |
| ORF22 | 29185-29577 | **-** | **GTP-binding protein** |
| ORF23 | 29574-30104 | **-** | **RNA polymerase ECF sigma factor** |
| ORF24 | 30363-30713 | **-** | Hypothetical protein |
| ORF25 | 30771-31517 | **-** | Hypothetical protein |
| ORF26 | 32080-32244 | **-** | Hypothetical protein |
| ORF27 | 32210-32713 | **-** | **Serine/threonine protein phosphatase** |
| ORF28 | 33093-33272 | **-** | Hypothetical protein |
| ORF29 | 33286-33519 | **-** | **5’-3’ exonuclease** |
| ORF30 | 33971-34864 | **-** | **ATP-binding protein** |
| ORF31 | 35630-36274 | **-** | **ATP-binding protein** |
| ORF32 | 36561-36728 | **-** | Hypothetical protein |
| ORF33 | 36725-37117 | **-** | Hypothetical protein |
| ORF34 | 37164-37340 | **-** | Hypothetical protein |
| ORF35 | 37350-37517 | **-** | Hypothetical protein |
| ORF36 | 37517-37636 | **-** | Hypothetical protein |
| ORF37 | 37701-37892 | **-** | Hypothetical protein |
| ORF38 | 37901-39709 | **-** | **DNA polymerase** |
| ORF39 | 39762-40184 | **-** | Hypothetical protein |
| ORF40 | 40186-40344 | **-** | Hypothetical protein |
| ORF41 | 40348-40632 | **-** | Hypothetical protein |
| ORF42 | 40634-40888 | **-** | Hypothetical protein |
| ORF43 | 40958-41167 | **-** | Hypothetical protein |
| ORF44 | 41262-41576 | **-** | Hypothetical protein |
| ORF45 | 41747-41914 | **-** | Hypothetical protein |
| ORF46 | 41911-42087 | **-** | Hypothetical protein |
| ORF47 | 42489-42665 | **-** | Hypothetical protein |
| ORF48 | 42675-42842 | **-** | Hypothetical protein |
| ORF49 | 42842-42961 | **-** | Hypothetical protein |
| ORF50 | 43026-43217 | **-** | Hypothetical protein |
| ORF51 | 43217-43582 | **-** | Hypothetical protein |
| ORF52 | 43591-43956 | **-** | **DNA polymerase** |
| ORF53 | 44563-44748 | **-** | Hypothetical protein |
| ORF54 | 44735-45064 | **-** | **NAD-dependent DNA ligase** |
| ORF55 | 45061-45246 | **-** | Hypothetical protein |
| ORF56 | 45227-45460 | **-** | Hypothetical protein |
| ORF57 | 45477-45752 | **-** | **Thymidylate synthase** |
| ORF58 | 45973-46182 | **-** | Hypothetical protein |
| ORF59 | 46182-46391 | **-** | Hypothetical protein |
| ORF60 | 46391-46588 | **-** | Hypothetical protein |
| ORF61 | 46585-46791 | **-** | Hypothetical protein |
| ORF62 | 46792-47325 | **-** | **Deoxycytidine triphosphate deaminase** |
| ORF63 | 47541-48914 | **-** | **Nucleotidyltransferase domain** |
| ORF64 | 48916-49251 | **-** | **DNA polymerase** |
| ORF65 | 49451-49711 | **-** | **Primase/ helicase** |
| ORF66 | 51103-51255 | **-** | Hypothetical protein |
| ORF67 | 53266-53379 | **-** | **Type I DNA polymerase** |
| ORF68 | 53635-53844 | **-** | Hypothetical protein |
| ORF69 | 53898-54035 | **-** | Hypothetical protein |
| ORF70 | 54080-54322 | **-** | Hypothetical protein |
| ORF71 | 54335-54583 | **-** | **ATP-grasp enzyme** |
| ORF72 | 55324-55461 | **-** | Hypothetical protein |
| ORF73 | 55506-55748 | **-** | Hypothetical protein |
| ORF74 | 55761-56009 | **-** | ATP-grasp enzyme |
| ORF75 | 56636-56773 | **-** | Hypothetical protein |
| ORF76 | 56818-57066 | **-** | Hypothetical protein |
| ORF77 | 57073-58167 | **-** | **ATP-grasp enzyme** |
| ORF78 | 61902-62309 | **-** | Hypothetical protein |
| ORF79 | 62529-62645 | **-** | Hypothetical protein |
| ORF80 | 63282-63689 | **-** | Hypothetical protein |
| ORF81 | 63909-64508 | **-** | Hypothetical protein |
| ORF82 | 65234-65596 | **-** | Hypothetical protein |
| ORF83 | 65599-65751 | **-** | Hypothetical protein |
| ORF84 | 65978-66442 | **-** | Hypothetical protein |
| ORF85 | 66442-66675 | **-** | Hypothetical protein |
| ORF86 | 67336-67584 | **-** | Hypothetical protein |
| ORF87 | 67586-67816 | **-** | Hypothetical protein |
| ORF88 | 67825-68136 | **-** | **Transcriptional regulator** |
| ORF89 | 69403-69621 | **-** | Hypothetical protein |
| ORF90 | 69622-69879 | **-** | Hypothetical protein |
| ORF91 | 70355-70651 | **-** | Hypothetical protein |
| ORF92 | 70851-71156 | **-** | Hypothetical protein |
| ORF93 | 71446-71619 | **-** | Hypothetical protein |
| ORF94 | 71622-72038 | **-** | Hypothetical protein |
| ORF95 | 74914-75279 | **-** | Hypothetical protein |
| ORF96 | 75473-75733 | **-** | Hypothetical protein |
| ORF97 | 75723-75989 | **-** | Hypothetical protein |
